# Three novel polymorphic microsatellite markers for the glaucoma locus GLC1B by datamining tetranucleotide repeats on chromosome 2p12-q12

**DOI:** 10.1590/S1415-47572009005000093

**Published:** 2009-12-01

**Authors:** Carlos Murga-Zamalloa, Maria Luisa Guevara-Fujita, Alejandro Estrada-Cuzcano, Ricardo Fujita

**Affiliations:** Centro de Genética y Biología Molecular, Facultad de Medicina, Universidad de San Martín de Porres, LimaPerú

**Keywords:** GLC1B, microsatellite polymorphic markers, tetranucleotide tandem repeat, gene mapping, glaucoma

## Abstract

In order to identify new markers around the glaucoma locus GLC1B as a tool to refine its critical region at 2p11.2-2q11.2, we searched the critical region sequence obtained from the UCSC database for tetranucleotide (GATA)n and (GTCT)n repeats of at least 10 units in length. Three out of four potential microsatellite loci were found to be polymorphic, heterozygosity ranging from 64.56% to 79.59%. The identified markers are useful not only for GLC1B locus but also for the study of other disease loci at 2p11.2-2q11.2, a region with scarcity of microsatellite markers.

Primary open angle glaucoma (POAG), an optic neuropathy, is one of the leading causes of blindness (prevalence 3-7% worldwide), although early detection makes it preventable (Wolfs *et al.*, 2000). POAG heritability is high and family studies revealed at least eight loci with only two causative genes identified so far (Challa, 2004). Locus *GLC1B* was mapped by linkage analysis in British families to a region of about 21 Mb flanked by markers *D2S176* and *D2S2161* on chromosome 2cen-q13 (Stoilova *et al.*, 1996). We reported a Peruvian family with POAG linked to the *GLC1B* region, with an unaffected member presenting the same haplotype as affected relatives, regarding markers D2S2264, *D2S1897*and *D2S176*, but not sharing the alleles at *D2S417* and *D2S2161* (Fujita *et al.*, 2002; Guevara-Fujita *et al.*, 2003). The segment delimited by D2S176 and *D2S2264* was discarded for *GLC1B*, thus narrowing down the locus critical region to about 15,5 Mb (Fig 1).

In order to refine the location of *GLC1B*, we sought markers between *D2S2264* and *D2S417* which were about 15 Mb apart. However, the reported microsatellite markers did not evenly cover this region, neither were informative in the family we were studying.

This prompted us to search for tetranucleotide repeat markers in the *GLC1B* region, since it is well recognized that tetranucleotide motifs present better allele separation and extra bands than dinucleotide. We searched the NCBI HUMAN GENOME DATASE for these motifs mapping at the segment of about 16 Mb between markers *D2S417* and *D2S2264* at 2p11.2-2q11.2. Primers flanking candidate markers were designed and their heterozygosity tested in healthy non-related individuals and in our previously reported POAG family. (Fujita 2002, Guevara-Fujita *et al.*, 2003). DNA was obtained from peripheral blood of a total of 106 healthy non-related volunteers using routine salting-out method.

Sequences from 2p11.2 and 2q11.2 (between *D2S417* and *D2S2264*) from NCBI BUILD 36.1 available from ‘The Human Genome Browser at UCSC' Genomic Library were searched for tetranucleotide repeats with (GATA)_n_ motifs at least 10 units long their flanking sequences, using BIOEDIT SEQUENCE ALIGNMENT EDITOR^®^ software. Selected sequences were analyzed using VECTOR NTI 8^®^ (Demo version) for primer design calculations. Reactions were performed in a volume of 10 μL, containing 50 ng of DNA, 2.5 mM each of dNTPs, 1 mM of each primer, 1 unit of Taq polymerase and 1 μL of 10X buffer with 1 μL of 10X MgCl_2_. PCR products were amplified on an Amplitron II Thermolyne thermocycler cycling conditions: 35 cycles of 94 °C for 30 s, optimal annealing temperature for 30 s and 72 °C for 30 s. PCR products were electrophoresed on 5 or 6% denaturing acrylamide gels, alleles subsequently revealed by silver staining. Allele sizes were determined by comparison with a pUC18 sequencing reaction product.

Four sequences with a (GATA)n motif were identified on the 15,5 Mb segment between 2p11.2 and 2q11.2. To assess heterozygosity, 106 individuals (212 chromosomes) were genotyped from a sample of Lima population, a heterogeneous admixture of South American native and Caucasian ancestries, with minor Asian and African contributions. Three of the identified loci turned out to be polymorphic (Table 1). Figure 1 shows the relative location of the new markers D2SCATTO3, D2SCATTO4, D2SCATTO2 and D2SCATTO1 and of the reference markers between *D2S216* and *D2S176* on the *GLC1B* locus region on chromosome 2. Table 1 also shows the optimal annealing temperature, allele frequencies, allele size and heterozygosity calculated for each new marker. Marker *D2SCATTO1* showed eight alleles ranging from 244 to 272 base pairs (bp) with heterozygosity of 75.15%, marker *D2SCATTO2* eight alleles ranging from 319 to 347 bp with heterozygosity of 79.59%, and marker *D2SCATTO3* three alleles ranging from 221 to 229 bp with heterozygosity of 64.56%. At D2SCATTO4 only one 291 bp product was obtained in the sample analyzed. Allele sizes of three of the new polymorphic markers allow for multi-loading, making genotyping easier and reducing lab work and time. Primer sequences, cytogenetic localization and allele sizes of each marker are deposited in the NCBI dbSNP (BUILD B131, D2SCATTO1 NCBI ss: 142466905; D2SCATTO2 NCBI ss: 142466907; D2SCATTO3 NCBI ss: 14246691; 2D2SCATTO4 NCBI ss:142466910).

These markers can be used not only for screening families with POAG populations (Wolfs *et al.*, 2000), but also in other genetic studies in region 2p11.2-q11.2 where loci for different diseases mapped. Examples are Amish Infantile Epilepsy Syndrome (MIM: 609056), Chronic Obstructive Pulmonary Disease with Severe Early-Onset (MIM: 606963), Achromatopsia 2 (MIM: 216900) and Ahnidrotic Ectodermal Dysplasia (MIM: 224900). The study of other diseases mapped the overlapping region such as Schizophrenia (MIM: 181500), Congenital Cataract (MIM: 607304), Combined Deficiency of Vitamin K-Dependent Clotting Factors (MIM: 277450), Ataxia-Telangiectasia (MIM: 208900), Congenital Pulmonary Alveolar Proteinosis (MIM: 178640) Nephronophthisis (MIM: 256100) could also benefit from the use of these markers.

## Figures and Tables

**Table 1:** Markers identifed in the present study, genomic location, allele sizes and frequencies, and primer sequences.

Markers	Cytogenetic location	Primers		Annealing temperature	Alleles (bp)	Frequency
		Forward	Reverse			
D2SCATTO3	2p11.2 (Chr2: 89399095-89399314 Mb)	GGTCCAATTCCCTGGAACCACCAG	GCCAGATAGCCAGTGGCAGGACC	62 °C	221	0.25
					225	0.45
					229	0.30

D2SCATTO4	2q11.2 (Chr2: 97104768-97105061 Mb)	GCACCAGGCTCTATCCTGCACC	GGGTTTCAGCTGTTTGTAACAGCC	60 °C	264	1.00

D2SCATTO2	2q11.2 (Chr2: 99437128-99437461 Mb)	TGTACTCCCTCTCCGGGGATC	GGGCCATACTGTGTTTACAGGAGC	60 °C	347	0.01
					343	0.02
					339	0.10
					335	0.19
					331	0.19
					327	0.32
					323	0.12
					319	0.04

DS2CATTO1	2q11.2 (Chr2: 100654172-1006544369 Mb)	ACAAAACTTAGCCGGGCATGG	CAATGAACCATCACAGTCAGGG	62 °C	272	0.04
					268	0.27
					264	0.39
					260	0.11
					256	0.06
					252	0.02
					248	0.03
					244	0.09

## References

[Challa2004] Challa P. (2004). Glaucoma genetics: Advancing new understandings of glaucoma pathogenesis. Int Ophthalmol Clin.

[ChungandGardiner1996] Chung E., Gardiner R.M., Adolph K.W. (1996). Mapping human diseases genes by linkage analysis. Methods in Molecular Genetics: Human Molecular Genetics.

[Fujitaetal2002] Fujita R., Guevara-Fujita M.L., Perez-Grossman R., Richards J. (2002). Cosegregation of glaucoma with locus GLC1B (2q12-q13) in a Peruvian family with heterogeneous onset and a recombination within the critical region. Abstracts of the Annual Meeting of The American Society of Human Genetics 71:A1570.

[Guevara-Fujitaetal2003] Guevara-Fujita M.L., Perez-Grossman R., Vargas E., Fujita R. (2003). Mapeo cromosómico y refinamiento de la localización de un en de glaucoma en 2q12-q13 en una familia Peruana: Avances hacia la clonación del gen GLC1B. Horizonte Médico.

[Stoilovaetal1996] Stoilova D., Child A., Trifan O.C., Crick R.P., Coakes R.L., Sarfarazi M. (1996). Localization of a locus (GLC1B) for Adult-Onset Primary Open Angle Glaucoma to the 2cen-q13 Region. Genomics.

[Wolfsetal2000] Wolfs R.C.W., Borger P.H., Ramrattan R.S., Klaver C.C.W., Hulsman C.A.A., Hofman A., Vingerling J.R., Hitchings R.A., de Jong P.T.V.M. (2000). Changing views on Open-Angle Glaucoma: Definitions and prevalences - The Rotterdam Study. Invest Ophthalmol Vis Sci.

